# Enhancing Quality Characteristics of Buckwheat (*Fagopyrum esculentum*) Flour: Effects of Germination and Nixtamalisation Processes

**DOI:** 10.1155/sci5/9935662

**Published:** 2025-12-30

**Authors:** Kazeem Koledoye Olatoye, Abiola Folakemi Olaniran, Oluwatobi Ibukun Adeyemo, Adeniyi Ayokanmi Koledade, Faith Oluwatoyosi Agaja, Oluwatobi Victoria Obayomi

**Affiliations:** ^1^ Department of Food Science and Technology, Faculty of Agriculture, Kwara State University, P.M.B 1530 Malete, Ilorin, Kwara State, Nigeria, kwasu.edu.ng; ^2^ Department of Food Science and Microbiology, College of Pure and Applied Sciences, Landmark University, P.M.B. 1001, Omu Aran, Nigeria, lmu.edu.ng

**Keywords:** buckwheat, digestibility, functional properties, germination, nixtamalisation

## Abstract

Buckwheat is a pseudo‐cereal with chemical, functional and application comparable to wheat but possesses higher antinutrient contents, which limit their digestibility and broader utilisation. The study investigated the effects of germination and nixtamalisation on the quality characteristics of buckwheat flour. Germination and nixtamalisation processes were carried out using limewater and potassium hydroxide. Whole, germinated buckwheat flour (GBW), organically nixtamalised buckwheat flour and synthetically nixtamalised buckwheat flour (SNBW) were produced and analysed for nutritional composition (proximate and mineral contents), antinutrients (phytate, oxalate, tannin and saponin) and antioxidant properties (phenol, flavonoids, ferric‐reducing antioxidant power and total antioxidant), physicochemical and functional properties (water absorption capacity [WAC], oil absorption capacity (OAC), swelling capacity (SC) and bulk density (BD), and data were analysed using ANOVA at *α*_0.05_. Germination and nixtamalisation processes significantly increased the moisture content (5.67%–8.67%; *p* < 0.05, ash (1.67%–4.30%; *p* value *p* < 0.05), crude protein (11.43%–14.91%; *p* < 0.05), fibre (10.20%–13.20%) and fat (5.0%–15.0%) but reduced the carbohydrate (61.52%–50.43%) of buckwheat flour. Protein digestibility of buckwheat flour was significantly improved (65.11%–78.14%). Similar trends were observed for the mineral content and antioxidant properties of the treated flours. The antinutritional properties of flour samples were reduced by both germination and nixtamalisation. Germinated buckwheat showed higher lightness (*L*), redness (*a*) and light intensity (*E*) compared with nixtamalised ones. Germination and nixtamalisation significantly influenced the pH, WAC, OAC, SC, solubility and bulk density of the buckwheat flour, which ranged between (4.94–8.91), (74.67–190.33 mL/g), (72.00–84.00 mL/g), (6.28–9.87 mL/g), (15.67–52.67 mL/g) and (0.8–0.85 g/mL), respectively. The application of germination and nixtamalisation processes significantly improves the protein digestibility, mineral content, and acidity of buckwheat flour as compared to whole buckwheat. Thus, these methods of processing have been proven to further enhance the qualitative attributes of buckwheat flour, promoting its expanded application in the food sector.

## 1. Introduction

Buckwheat is an underexploited rich source of nutrients and phytochemical compounds. It is an ancient pseudo‐cereal crop under the Polygonaceae family and genus *Fagopyrum,* which occupies a crucial part of the human diet, consumed globally [1–3]. It is a rich source of protein (20% or more of the daily value [DV]), dietary fibre, B vitamins and several dietary minerals, with a high content (47%–65% DV) in niacin, magnesium, manganese and phosphorus [4]. More importantly, buckwheat contains no gluten and, as such, can be consumed easily by people with gluten‐related disorders, such as coeliac disease, nonceliac gluten sensitivity or dermatitis herpetiformis [4]. In addition, its contents of phytonutrients and antioxidant properties were acknowledged against diseases such as cancer and heart attacks [5]. In comparison with similar food grains such as conventional cereals, buckwheat possesses a very low glycaemic index, and thus, its carbohydrate content is absorbed slowly into the bloodstream and provides the body with a steady flow of energy [5]. By preventing a sudden spike in blood sugar, this nutritious seed helps with diabetes management and may improve insulin resistance. Nonetheless, protein digestibility was reportedly low for a buckwheat‐based diet, perhaps due to the presence of antinutrients such as protease inhibitors, phytate and tannin [6]. Antinutrients are natural compounds that obstruct the digestion and absorption of nutrients [7]. This calls for applications of various methods with proven records of reducing the antinutrient contents in similar food materials, particularly cereal, including buckwheat. Two examples of such methods are germination and nixtamalisation. Germination is the process by which a seed grows after a period of dormancy sequel to the right environmentally friendly circumstances leading to water uptake, enzyme activation and breakdown of stored nutrients [8]. This process increases the availability of nutrients in seeds, grains and legumes [9]. Germination may take a few days or more than a week. During this process, several changes do occur within the grain which may lead to the degradation of antinutrients such as phytate and protease inhibitors. Studies have established the benefits of germination in improving the nutritional value of cereals since it has been found to improve the content of vitamins such as tocopherols (α‐, β‐ and γ‐tocopherols), riboflavin (Vitamin B_2_) and total niacin (Vitamin B_3_) in cereals and legumes. The method removes antinutritional factors such as phytic acid and tannins that chelate minerals, thereby improving the availability of iron, zinc and calcium. Activation of enzymes in germination causes the breakdown of complex carbohydrates and proteins through hydrolysis, making them easier to digest [10, 11].

Nixtamalisation is a process whereby a mature dried cereal grain such as maize (corn) is cooked and soaked in an alkaline solution. The process can also be done using a natural source of alkaline (limewater) or synthetically, using potassium hydroxide (KOH). Both were reportedly safe and effective in removing antinutrients from cereals. The nixtamalisation process can be completed within a shorter time than the germination process [9]. Innovative applications of nixtamalisation have been explored with other cereals, such as oats, to produce tortillas with improved nutritional and sensory properties. It has been shown that nixtamalisation improves the nutritional quality of maize tortillas, including increased calcium content and protein quality. In addition, it has been found to be effective in the decontamination of aflatoxin in maize by as much as 70%, consequently enhancing food safety [12, 13]. These advances emphasise the effectiveness and flexibility of germination and nixtamalisation in improvement of cereal nutritional value and resulting in enhanced health and food security. Therefore, this research was designed to compare the performances of these processing methods relative to their effects on the chemical, physicochemical and functional properties of buckwheat flour.

## 2. Materials and Methods

### 2.1. Sample Collection and Preparation

Buckwheat grains (*Fagopyrum esculentum)* were obtained from a local market in Ilorin, Kwara State, Nigeria. All chemicals/reagents such as HCl_(aq)_, NaOH_(aq)_, toluene phosphate buffer, trichloroacetic acid (TCA) and enzymes (pancreatin, pepsin) used in this study were of good analytical grades and were manufactured by Antech Diagnostic Products, United Kingdom; East Anglia Chemicals, Hadleigh Ipswich Suffolk; W. A. Taylor & Co. Inc. Baltimor MD USA, as obtained from Chemistry, Food Science and Technology and Microbiology Laboratory Departments, Kwara State University, Malete.

### 2.2. Experimental Design

Completely randomised design (CRD) was used. This involves 4 treatments, (whole buckwheat, germinated bulk wheat, naturally nixtamalised buckwheat and synthetically nixtamalised buckwheat) and 3 replicates (*n* = 3), making a total of 12 samples. In order to minimise experimental bias, treatments were randomly assigned and analysed in triplicates. Standardised protocols and calibrated equipment were used throughout. Samples were coded to ensure blinded analysis and processed under uniform conditions. Measurement order was balanced to avoid time‐related bias.

### 2.3. Production of Germinated Buckwheat Flour (GBW)

The method of Devarajan et al. [14] was used to germinate buckwheat grains and process them into flour. Cleaning was done by applying an aspirator and sieving to remove foreign seeds and saw dust. The cleaned 10 kg of buckwheat grains were washed and steeped in excess water for 12 h. The water was changed once or twice during the steeping period. At the end of the steeping period, the grains were washed again and kept for germination for 72 h. Steeped buckwheat grains were spread on about 2‐3 inches thick bed, then covered with another moist cloth and placed near a source of light to provide warmth and maintain the optimal temperature range (30°C ± 2°C) and relative humidity of 60% ± 2%. During germination, water was sprinkled daily to keep the sprouts moist. After germination, the sprouts were dried in a cabinet dryer at 65°C ± 2°C for 5‐6 h and then the sprouts were milled. Grinding of the green malt was done using a burr mill (SKU: GE779HA3WBN9ANAFAMZ made in China). Malt flour was sieved through 0.25 mm (mesh size) and stored at room temperature.

### 2.4. Production of Naturally Nixtamalised Buckwheat Flour (NNBW), Using Limewater

The method described by Guzmán‐de‐Peña [15] was used. The grain sample (10 kg) was steeped in water for 10 min, drained and then poured into the boiling solution containing (limewater) and allowed to cook for 6 min. After cooking, the dehulled grain was allowed to steam for 10 min then drained and washed in running water to achieve a clean grain which will undergo drying in a cabinet dryer at a temperature of 65°C ± 2°C for 6 h. Dried grain was milled, and the flour was sieved to achieve a smooth texture and then stored at room temperature.

### 2.5. Production of Synthetically Nixtamalised Buckwheat Flour (SNBW), Using Potassium Hydroxide (KOH)

Modified methods of Guzmán‐de‐Peña [15] and Odukoya et al. [16] were used. The grain (10 kg) was steeped in 15 L of water and changed at three intervals. About 1 M potassium hydroxide (KOH) was prepared by dissolving 56 g of KOH pellets in distilled water (250 mL); thereafter, the solution was made up to 1000 mL with distilled water and allowed to boil before the buckwheat grain was added. It was then allowed to cook for 6 min, and the cooked grain was washed and dried in a cabinet dryer at 65°C ± 2°C for 6 h. Then, grain was milled, and the flour was sieved, then stored at room temperature. Potassium hydroxide is an ideal cooking ingredient for cereal nixtamalisation without bringing loss of dietary minerals, and additionally, washing with clean water will remove remnants of KOH after nixtamalisation [16].

### 2.6. Production of Whole Buckwheat Flour (WBF)

Whole buckwheat grain was cleaned by application of an aspirator and sieving for the removal of foreign seed, saw and dust. The cleaned buckwheat grain was dried in a cabinet dryer at 65°C ± 2°C, milled, sieved and stored at room temperature.

### 2.7. Analyses

#### 2.7.1. Determination of Proximate Composition and Energy Values of Buckwheat Flour

Proximate compositions of flour samples were determined by the official method of analysis AOAC [17]. The samples were analysed for moisture, protein, fat, ash, fibre and available carbohydrate (by difference). The energy value was determined by the method described by MacLean [18]. The available energy value of the food sample was calculated as follows:

(1) Energy value in kilojoules per 100 g=% carbohydrate×171737+% protein×+% fat×.

#### 2.7.2. Determination of the Mineral Composition of Buckwheat Flour

Minerals such as iron, zinc, manganese and calcium were estimated according to the respective method as described in AOAC [17] using an Atomic Absorption Spectrophotometer (Varian, AA240, Victoria, Australia). The standard curve was prepared by running samples of known strength through an atomic absorption spectrophotometer. The mineral contents of unknown samples were estimated by using the respective standard curve prepared for each mineral.

#### 2.7.3. Determination of Antioxidant Properties of Buckwheat Flour

The concentration of phenolic in the flour sample was determined using the spectrophotometric method [19]. Total phenolic content was determined using a modified procedure described by Olaniran et al. [19]. Each sample (0.1 g/mL in water) was mixed with 5.9‐mL distilled water, and 1.0 mL of the diluted extract was mixed with 1.0 Folin–Ciocalteu reagent. The mixture was allowed to stand for 2–5 min, and 2 mL for 20% (w/v) Na_2_CO_3_ was added. After 30 min of rigorous mixing with a vortex mixer, absorbance was taken 2 t 725 nm in a spectrophotometer. The result was expressed as gallic acid equivalent (GAE) using a calibration curve with gallic acid as standard (100 mg/mL).

The total antioxidant activity of the extract (filtrate from 5 g of flour in 100 mL of solvents; 95% ethanol and water at a ratio 1:1) that had been stirred using a magnetic stirrer for 30 min at room temperature. The absorbance was 517 nm measured by UV‐spectrophotometry with the purple‐coloured solution of DPPH radical. About 0.5 mL of DPPH solution in 95% ethanol was prepared, and 0.5 mL of the solution was mixed with 1.5 mL of the sample solutions in 95% ethanol. A decrease in DPPH solution absorbance indicated an increase in the DPPH radical scavenging activity, which was calculated. DPPH radical tests used for assessment of radical scavenging activity of the samples and the resulting antioxidant activities were expressed as IC50 (mg/mL) value (concentration of sample required to scavenge 50% of free radicals). A lower IC50 value indicated greater antioxidant activity [20]. The reducing property of samples was determined by assessing the ability of the sample to reduce ferric chloride (FeCl_3_) solution as described by Pulido et al. [21]. Exactly 2.5‐mL aliquot volume was mixed with 2.5 mL of 200 mM sodium phosphate buffer (pH 6.6) and 2.5 mL of 1% potassium ferricyanide. The mixture was incubated at 50°C for 20 min, and 2.5 mL of 10% TCA was added. The mixture was centrifuged at 805 g for 10 min. A volume of 1 mL of different concentrations (50, 100, 150 and 200 μL) of the supernatant was mixed with an equal volume of water and 1 mL of 0.1% ferric chloride. The absorbance was measured at 700 nm in the spectrophotometer (Jenway 6305) after the solution had stood for 30 min. A graph of absorbance versus concentration was plotted to observe the reducing power where higher absorbance values indicated a higher reducing power.

Total flavonoid content was measured according to a colourimetric assay [22]. Total flavonoid content was determined by aluminium chloride colourimetric assay. The quercetin standard solution in concentration 30, 40, 50, 60, 70, 80, 90 and 100 μg/mL was prepared in 95% ethanol. About 50 μL of extracts (1 mg/mL) or standard solution was mixed with 10 μL of 10% the aluminium chloride solution and followed by 150 μL of 96% ethanol. About 10 μL of 1‐M sodium acetate was added to the mixture in 96‐well plate. Ninety‐six percent ethanol was used as reagent blank. All reagents were mixed and incubated for 40 min at room temperature in the dark. Absorbance reading was taken at 415 nm by using a microplate reader (Versamax Microplate Reader, USA). Total flavonoid contents were estimated and expressed as mg quercetin equivalents (QE) per g of extract.

#### 2.7.4. Determination of Antinutrients of Buckwheat Flour

The method of Olatoye and Arueya [23] was employed to determine the phytate content. Determination of oxalate was carried out according to Day and Underwood [24], while the Folin–Denis colourimetric method described by Kirk and Sawyer [25] was employed to determine tannin content.

#### 2.7.5. Determination of In Vitro Protein Digestibility of Buckwheat Flour

In vitro digestibility of protein was done, 1 g of the sample was added to 15 mL (0.1 M) HCl containing 1.5 mg pepsin and incubated for 3 h at 37°C; 7.5 mL (0.2 M) NaOH was then used to neutralise the obtained suspension which was then treated with 4 mg of pancreatin in 7.5 mL (0.2 M) phosphate buffer. Toluene was added to prevent microbial growth, and the mixture was shaken and incubated for another 24 h. After the incubation period, 10 mL of 10% TCA was added and centrifuged for 20 min to remove the undigested protein and larger peptides from the sample. The Kjeldahl method [26] was used to estimate the protein in supernatant. Nitrogen contents in a blank sample were also estimated. Protein digestibility was calculated by the following formula.

(2) Protein digestibility %=Nitrogen in supernatant – Nitrogen in blank×100.

##### 2.7.5.1. Nitrogen in Sample

The Jones factor (6.25) was used to calculate protein content by multiplying the total nitrogen content of a food by 6.25. Validation was done by replicating findings, ensuring specificity and sensitivity. Values of protein digestibility of samples were compared with specific protein digestibility of Zein (corn) (65.10%), a cereal, in line with INFOGEST in vitro digestion protocol, as reported by Sousa et al. [27].

#### 2.7.6. Determination of Physicochemical Properties of Buckwheat Flour

The pH of the buckwheat flour was measured using a pH 211 microprocessor pH metre (Hanna, Woonsocket, RI, USA). Total titratable acidity (TTA) was determined according to Olaniran et al. [19].

#### 2.7.7. Determination of Physical Colour of Buckwheat Flour

The physical colour of the buckwheat flour was quantitatively determined with the aid of a handheld colourimeter (Tec PCM/PSMTMI Colour Metre), as described by Babajide and Odulate [28]. The instrument was first standardised against a white reference plate. Thereafter, five measurements were taken for each sample, and direct measurement of three colour features of *L* ^∗^ (lightness), *a* ^∗^ (red‐green component) and *b* ^∗^ (yellow‐blue component) was done by the colourimeter.

Total colour difference (TCD) represented as ΔE *was* determined using the following equation.

(3) ΔE=ΔL2+Δa2+Δb2 12/,

where (Δ*L*) represents the change in lightness; (Δ*a*) represents the change in the red‐green component and (Δ*b*) represents the change in the yellow‐blue component of flour samples.

#### 2.7.8. Determination of Functional Properties of Buckwheat Flour

The bulk density (BD), water and oil absorption capacities were analysed using Olaniran and Abiose [29], while the procedure of Oladele and Aina [30] was used for the swelling capacity determination.

#### 2.7.9. Statistical Analysis

All data obtained from all analyses of each sample carried out in triplicates were analysed with the aid of a one‐way analysis of variance (ANOVA). Separations of means were done by Duncan’s multiple range test (DMRT) using the Statistical Package for Social Science (SPSS) IBM Version 23.0 package. Significance was accepted at *p* < 0.05.

## 3. Results and Discussion

### 3.1. Influence of Processing Methods on Proximate Composition of Buckwheat Flour

From the statistically analysed data using ANOVA presented in Table 1, the proximate composition of buckwheat flour significantly varied at *p* < 0.05. It was observed that GBW had the highest moisture content (8.67%), while SNBW (5.43%) had the lowest moisture content. The moisture content of all samples is < 14% which is good for storage stability [20]. However, wetting and soaking processes might have contributed to the increase in moisture content of GBW and NNBW compared to the control sample. With regard to crude protein content, Sample NNBW (14.91%) was the highest, in comparison to control WBW (13.18%) and other samples. GWB (11.43%) had the lowest protein content compared to the initial protein content of the flour (WBW) (13%). This observation could be ascribed to the concentration effect on nitrogen content, while a slight decrease in protein content of germinated samples may be due to the utilisation of released amino acids as a precursor for the synthesis during sprouting [31]. Nixtamalisation might reduce the nonproteinaceous compounds such as starch and other carbohydrates, so the ratio of protein may relatively increase.

**Table 1:** Proximate composition of buckwheat flour as influenced by processing methods.

Samples	Moisture (%)	Crude protein (%)	Ash (%)	Crude fibre (%)	Crude fat (%)	Carbohydrate (%)
WBW	5.67 ± 0.29^c^	13.18 ± 0.04^b^	1.67 ± 0.29^d^	10.20 ± 0.03^c^	7.77 ± 0.46^c^	61.52 ± 0.54^a^
GBW	8.67 ± 0.29^a^	11.43 ± 0.04^d^	3.50 ± 0.50^b^	13.20 ± 0.03^a^	12.67 ± 2.08^b^	50.54 ± 2.00^c^
NNBW	7.90 ± 0.10^b^	14.91 ± 0.05^a^	1.83 ± 0.58^c^	12.30 ± 0.02^b^	5.00 ± 0.00^d^	58.05 ± 0.54^b^
SNBW	5.43 ± 0.51^d^	11.84 ± 0.03^c^	4.30 ± 0.17^a^	13.00 ± 0.00^a^	15.00 ± 0.00^a^	50.43 ± 0.66^c^

*Note:* Sample codes: NNBW: naturally nixtamalised buckwheat flour. *n* = 3. Means in the same column with the same superscripts are not significantly different (*p* > 0.05).

Abbreviations: GBW, germinated buckwheat flour; SNBW, synthetically nixtamalised buckwheat flour; WBW, whole buckwheat flour.

Germination and nixtamalisation significantly increased the ash content of buckwheat with values varying from 1.67% to 4.30% with SNBW having the highest. Synthetically nixtamalised cereals have been reported to absorb more minerals from alkaline solutions, resulting in increase in the amount of inorganic residue retained during processing, thus leading to increase in the ash content [32]. Significant difference (*p* < 0.05) and slight increase in fibre content were observed in samples with applications of germination and nixtamalisation compared to the untreated buckwheat. The observed increase in the fibre content of processed flour may be due to breaking down of the grain’s outer layers, making the fibre more concerted and bioavailable. In addition, the processes reduce soluble components such as nonfibrous polysaccharides and starch, and eventually increase the relative fraction of fibre in the final product [33–35]. SNBW possessed the highest fat content (15%), while NNBW had the lowest value (5%). The observed increase in fat may be due to the degradation of by‐products of the grains during germination by hydrolytic enzymes such as lipases, amylases and proteases. These enzymes primarily function to supply energy and building blocks for the developing seedling by dissolving stored proteins, lipids and carbohydrates into simpler molecules [36]. However, the reduction of fat content due to organic nixtamalisation was earlier documented [37]. The organic nixtamalisation process has been reported to reduce fat content due to the alkaline pH in the nixtamalised buckwheat, which was lost due to the hydrolysis of lipid ester linkages and the free fatty acids. Thus, the result of the study with the lowest crude fat content in NNBW conforms to previous documentations [37, 38]. Reduced crude fat content in buckwheat flour can be advantageous in terms of extending shelf life [39]. With regards to carbohydrates, WBW had the highest carbohydrate content of 61.52%, while SNBW had the lowest value of 50.43%. The decrease in carbohydrate values in the germinated and nixtamalised samples can be a result of hydrolyses of carbohydrates and consequent production of various intermediate and coproducts [40].

### 3.2. Effect of Processing Methods on the Mineral Composition of Buckwheat Flour

Results from Table 2 revealed that the minerals Mg, Ca, Na and K were found in detectable amounts but with some variations. This agrees with the daily mineral and calorie requirement for individuals. Magnesium content increased in germinated and nixtamalised samples relative to GBW and WBW. The observed increase in magnesium could be associated with a reduction of antinutrients [41]. Similarly, the two methods increased the sodium content. In terms of potassium, the germinated sample had a higher value than the control, while nixtamalised samples had lower values. The sodium content ranged from 154.04% to 189.23% with SNBW having the highest value, while WBW had the lowest value. The low content of sodium in WBW makes it a preferred option for hypertensive individuals, while the higher potassium contents in GBW suggest its applicability in maintaining fluid balance. This is in line with Akpoghelie et al. [42], who reported that Na is essential for normal heart and muscle function. K helps in muscle contraction and maintains fluid balance and normal blood pressure. The presence of K and Na is an added advantage. Calcium contents of GBW and NNBW were higher than the control, while SNBW had the lowest value. Nixtamalisation has been reported to promote an increase in the calcium contents of the buckwheat flours due to the absorption of calcium ions, attributed to the utilisation of limewater alkaline during the process [41, 43]. The mineral Ca is well‐known for its key role in bone health. It also helps to maintain heart rhythm and muscle function. A general survey revealed that Ca has been recommended for controlling Mg, P and K levels in the blood circulation. Calcium plays an important role in blood clotting. The concentration of calcium found in this study reveals that the consumption of pretreated buckwheat products may increase the calcium level in the blood, as reported by Akpoghelie et al. [42].

**Table 2:** Effect of processing methods on the mineral contents of buckwheat flour.

Samples	Mg (mg/g)	Na (mg/g)	K (mg/g)	Ca (mg/g)
WBW	130.26 ± 0.17^d^	154.02 ± 0.03^d^	200.02 ± 0.01^b^	286.59 ± 0.01^c^
GBW	134.01 ± 0.07^c^	167.54 ± 0.03^c^	239.45 ± 0.08^a^	301.21 ± 0.02^b^
NNBW	146.57 ± 0.01^a^	169.55 ± 0.06^b^	180.24 ± 0.02^d^	312.12 ± 0.05^a^
SNBW	136.22 ± 0.04^b^	189.23 ± 0.11^a^	195.54 ± 0.02^c^	272.96 ± 0.04^d^

*Note:* Sample codes: NNBW: naturally nixtamalised buckwheat flour. *n* = 3. Means in the same column with the same superscripts are not significantly different (*p* > 0.05).

Abbreviations: GBW: germinated buckwheat flour, SNBW: synthetically nixtamalised buckwheat flour; WBW: whole buckwheat flour.

### 3.3. Effect of Processing Methods on the Antioxidant Properties of Buckwheat Flour

Table 3 shows the antioxidant activities of buckwheat flour as influenced by the processing methods used in this study. The values of phenolic contents ranged from 2.52 to 5.82 mg GAE/g with Sample WBW having the highest content (5.82 mg/g), while Sample GBW had the lowest (2.52 mg GAE/g). There was no significant (*p* > 0.05) difference among Samples GBW (2.52 mg GAE/g) and SNBW (2.80 mg GAE/g. The decrease in the total phenolic content was similar to observations of Rajeswari et al. [44] and Abioye et al. [45], who carried out research on the impact of alkaline cooking on the proximate, phenolics and antioxidant activities of foxtail millet. Nixtamalisation and germination recorded a reduction in total phenolic when compared to whole buckwheat. This may be due to some leaching into the soaking water and sprouting process [43]. In terms of flavonoid content, Sample GBW had the highest value (2.20 mg QE/g), while NNBW had the lowest value (1.37 mg QE/g). The flavonoid content of the buckwheat flour was significantly (*p* < 0.05) increased by applying germination and limewater nixtamalisation processing procedures. The highest flavonoid content value found in the flour made from germinated buckwheat was in line with other studies conducted on the effects of germination on cereal or grains [46, 47]. Their results have been linked to the biochemical metabolic processes of seeds during germination, which could lead to the production of flavonoids and anthocyanins, two secondary plant metabolites. Flavonoids function by chelating free radicals or scavenging them, as well as by protecting against oxidative stress [48]. The antioxidant activity of germinated buckwheat can be significantly increased through increasing the flavonoids. Therefore, compared to ungerminated buckwheat flour samples, GBW showed higher flavonoid value and antioxidant activity, thus making it an advantageous natural source of flavonoids. Moreover, germinated buckwheat shows itself as a prospective functional meal for enhancing health [49]. Studies have also shown that an increase in flavonoids and antioxidant activity can lead to significant improvement in the antioxidant activities of germinated and nixtamalised buckwheat flour. Thus, boosting the nutritional value and antioxidant activities compared to unprocessed buckwheat [34]. Again, it was observed that the FRAP content of germinated and nixtamalised samples had higher values than control samples, with values ranging from 1.69 to 2.86 mmol Fe^2+^/g. The increase in FRAP may be due to liberation of bound polyphenols during germination and nixtamalisation processes. It has been reported that samples with higher reducing power usually have better abilities to donate electrons and free radicals to form stable substances, thereby interrupting the free radical chain reactions [47]. A similar trend was also observed in the values of total antioxidants. GBW had the highest total antioxidant capacity (0.28 μg/mL IC50), while the WBW possessed the lowest (0.17 μg/mL IC50). Both germination and nixtamalisation enhanced the antioxidant capacity of buckwheat flour. The presence of hydroxyl groups in the chemical structure of phenolic compounds, which can supply the required component as a radical scavenger, may be the cause of the high scavenging property of germination [46]. However, WBW flour possessed the highest value of phenol (5.82 mg/g), while GBW had the lowest (2.52 mg/g). This showed that buckwheat in its unprocessed form is a reservoir of phenol which reduces when subjected to processing. This result follows Olatoye and Arueya [40] that processing may lead to the loss of free phenolic substances.

**Table 3:** Effect of processing methods on the antioxidant properties of buckwheat flour.

Samples	Phenol (mg/GAE/g)	Flavonoid (mg QE/g)	FRAP (mmol Fe^2+^/g)	Total antioxidant (μg/mL IC50)
WBW	5.82 ± 0.19^a^	0.54 ± 0.03^c^	1.69 ± 0.20^d^	0.17 ± 0.02^d^
GBW	2.52 ± 0.18^d^	2.20 ± 0.07^a^	2.86 ± 0.21^a^	0.28 ± 0.02^a^
NNBW	3.31 ± 0.19^b^	1.37 ± 0.07^b^	2.40 ± 0.40^b^	0.23 ± 0.01^b^
SNBW	2.80 ± 0.06^c^	0.43 ± 0.02^d^	1.98 ± 0.04^c^	0.19 ± 0.00^c^

*Note:* Sample codes: NNBW: naturally nixtamalised buckwheat flour. *n* = 3. Means in the same column with the same superscripts are not significantly different (*p* > 0.05).

Abbreviations: GBW, germinated buckwheat flour; SNBW, synthetically nixtamalised buckwheat flour; WBW, whole buckwheat flour.

### 3.4. Effect of Processing Methods on the Antinutrient Properties of Buckwheat Flour

The result of the antinutritional content of the buckwheat flour samples, as influenced by processing methods, is presented in Table 4. There was a significant decrease in the phytate content in processed buckwheat flour from 0.4 to 0.29 mg/100 g. A similar trend was observed for the contents of tannin (0.81–0.29 mg/100 g) and saponin (0.75–0.38 mg/100 g. The results revealed that the application of germination and nixtamalisation processes reduced the antinutrient contents (phytate, tannin and saponin) of buckwheat flour compared to control (WBW) in this study. However, the oxalate content of samples ranged between 0.14 and 0.20 mg/100 g, with GBW being the highest and SNBW the lowest. The effect of these processing methods on the oxalate content was marginally lower than what was observed for other antinutrients in this study. Different processing methods such as soaking, germination, nixtamalisation, hot extrusion, cooking and sprouting have been reported to reduce antioxidants in food materials [40]. During germination, changes occurred within the grain that led to the degradation of antinutrients such as phytate and protease inhibitors. Luo et al. [50] reported that germination had been shown to reduce phytate by 37%–81% in various types of grains and legumes. Nixtamalisation has been reported to reduce antinutrient levels which may improve the bioavailability of calcium, iron and zinc as it tends to form complexes with these minerals [39]. Also, germination may enhance the minerals bioavailability of the buckwheat flour samples due to reduction of antinutrients responsible for binding minerals [40]. The application of germination and nixtamalisation reduced antinutrient content generally in these buckwheat flours and may therefore improve the availability of ions in the product [34].

**Table 4:** Effect of processing methods on the antinutrient properties of buckwheat flour.

Samples	Phytate (mg/100 g)	Tannin (mg/100 g)	Saponin (mg/100 g)	Oxalate (mg/100 g)
WBW	0.45 ± 0.08^a^	0.81 ± 0.04^a^	0.75 ± 0.04^a^	0.20 ± 0.05^a^
GBW	0.38 ± 0.02^b^	0.71 ± 0.03^b^	0.55 ± 0.05^b^	0.16 ± 0.07^b^
NNBW	0.35 ± 0.04^bc^	0.70 ± 0.03^b^	0.46 ± 0.05^c^	0.15 ± 0.02^bc^
SNBW	0.29 ± 0.01^c^	0.29 ± 0.02^c^	0.38 ± 0.01^d^	0.14 ± 0.02^c^

*Note:* Sample codes: NNBW: naturally nixtamalised buckwheat flour. *n* = 3. Means in the same column with the same superscripts are not significantly different (*p* > 0.05).

Abbreviations: GBW, germinated buckwheat flour; SNBW, synthetically nixtamalised buckwheat flour; WBW, whole buckwheat flour.

### 3.5. In Vitro Protein Digestibility of Buckwheat Flour as Influenced by Processing Methods

Application of germination and nixtamalisation significantly influenced (*p* < 0.05) the in vitro protein digestibility value of buckwheat flour (Table 5). The GBW sample possessed the highest (78.14%) value, followed by NNBW (77.04%) and SNBW (70.53%), which were also higher than that of WBW (65.11). With exception of WBW, these values are higher than (65.10) the in vitro protein digestibility value for Zein (corn), a reference cereal [27]. These findings also agreed with Hassan et al. [43], who reported low protein digestibility in WBW, which was reportedly due to the presence of antinutrients such as protease inhibitors, phytate and tannin, which are natural compounds that interfere with the digestion and absorption of nutrients. Protein digestibility increased as the tannins’ compound formation with proteins decreased during nixtamalisation [43]. Germination has been documented to improve the hydrolysis of proteins and increase peptides and amino acids availability [47]. The initiation of dormant enzymes which occur during germination led to significant modifications in the nutritional and biochemical characteristics of buckwheat flour. This may be due to the rapid degradation α‐ and β‐amylases, while other enzymes break down cell walls and enhance the bioaccessibility of nutrients [51]. Nixtamalisation has also been reported as an effective processing technique for increasing protein digestibility in food products by significantly reducing the tannin content of the cereals. Studies [52, 53] reported that nixtamalisation reduced 96 per cent of condensed tannins in sorghum, resulting in increased protein digestibility due to a reduction in the formation of tannin–protein complexes in condensed tannins that bind protein in sorghum, thus depolymerising condensed tannins and breaking protein–tannin complexes.

**Table 5:** In vitro protein digestibility of buckwheat flour as influenced by processing methods.

Samples	Protein digestibility (%)
WBW	65.11 ± 0.14^d^
GBW	78.14 ± 0.46^a^
NNBW	77.04 ± 0.17^b^
SNBW	70.53 ± 0.10^c^
Zein (corn)	65.10 ± 0.00^d^

*Note:* Sample codes: NNBW: naturally nixtamalised buckwheat flour; Zein (corn) is a reference cereal, according to INFOGEST in vitro digestion protocol, as reported by Sousa et al. [27]. *n* = 3.

Abbreviations: GBW, germinated buckwheat flour; SNBW, synthetically nixtamalised buckwheat flour; WBW, whole buckwheat flour.

^∗^Means in the same column with the same superscripts are not significantly different (*p* > 0.05).

### 3.6. Influence of Processing Methods on the Physicochemical Properties of Buckwheat Flour

TTA and pH were investigated, and data are presented in Table 6.

**Table 6:** Influence of processing methods on the physicochemical properties of buckwheat flour.

Samples	TTA (mg/100 g)	pH
WBW	0.47 ± 0.02^b^	5.86 ± 0.01^c^
GBW	0.20 ± 0.02^c^	5.98 ± 0.01^b^
NNBW	0.54 ± 0.04^a^	4.94 ± 0.01^d^
SNBW	0.14 ± 0.00^d^	8.91 ± 0.01^a^

*Note:* Sample codes: NNBW: naturally nixtamalised buckwheat flour. *n* = 3.

Abbreviations: GBW, germinated buckwheat flour; SNBW, synthetically nixtamalised buckwheat flour; WBW, whole buckwheat flour. Means in the same column with the same superscripts are not significantly different (*p* > 0.05).

The TTA values of buckwheat flour samples ranged from 0.14 mg/100 g to 0.54 mg/100 g, with NNBW being the highest and SNBW the lowest. The TTA value of NNBW can contribute to the inhibition of microbial growth, which may prolong the shelf life of items containing this flour [54]. The increased TTA can be traced to the use of lime in NNBW processing. Contrarily, the lowest TTA value in SNBW flour samples could be attributed to the alkalinity used in its processing. An alkaline solution used in the chemical processes applied to treat this flour might have reduced its acidity. The effect could be a subtle flavour, where a strong acidic flavour is not desired. Strong flavour and long shelf life are desirable qualities for organic and natural food products, and this kind of flour proved suitable for such applications [19]. The pH values ranged from 4.94 to 8.91, with SNBW being the highest and NNBW the lowest. This inferred that NNBW was the most acidic sample under investigation, while SNBW was the least. The pH values exhibited an inverse relationship with the TTA values of the samples. Therefore, the choice of buckwheat processing techniques should be based on desired factors such as long shelf life, self‐sanitary ability and mild or strong flavour requirement [54]. Many microbes find it difficult to survive in environments with low pH and high acidity, which lengthens the storage life [55]. Thus, various processing techniques affected the buckwheat flour’s acidity and general qualities.

### 3.7. Effect of Processing Methods on the Colour Attributes of Buckwheat Flour

The degree of product’s lightness (*L*), redness (*a*), yellowness (*b*) and light of intensity (Δ*L*) of buckwheat flour as influenced by different processing methods are presented in Table 7. The *L* ^∗^ values ranged between 60.70 and 68.51, with NNBW being the highest and SNBW the lowest. This result is in line with Qin et al. [56], who reported on the product’s lightness. It can be inferred that the nixtamalisation of buckwheat groat made the flour whiter than both germinated and whole bulk wheat. This can be a result of the effects of soaking water together with the lime or alkaline contents in NNBW and SNBW, respectively. The *a* ^∗^ values ranged between 1.63 and 3.40, with GBW being the highest and SNBW the lowest. This implied that GBW possessed the highest degree of redness among all the samples, while SNBW the lowest. The redness could be associated with various biochemical and enzymatic activities occurring during germination. However, red‐coloured flour is not desirable in most pastry applications. Thus, SNBW may find a better application as it tends to be whiter but not red. The *b* ^∗^ values ranged between 10.97 and 15.77, with SNBW being the highest and GBW the lowest. With regard to the *b* ^∗^ value, the positive *b* ^∗^ value of SNBW (15.77%) indicated the presence of yellow components in starch and flour. Additionally, the higher *b* ^∗^ value of flour was reported by the authors in [57] to be an indication of the presence of higher ash content, and the present study concurs with the findings. Lastly, the light intensity (Δ*L*‐values) ranged between 41.67 and 49.48, with NNBW being the highest and SNBW the lowest. It followed that the level of light intensity was enhanced by natural and synthetic nixtamalisation processes. This result agreed with earlier studies. The colour of nixtamalised corn was reported to be enhanced compared to the control. This was attributed to water diffusion during the processing, resulting in improved values of Δ*E* (TCD) [58]. Colour development in lime‐treated products could result from the lime, thereby contributing to the intensity of colour which is closely related to the lime concentration. An increase in the concentration of lime has been reported to lead to a more yellowish product due to absorption in *Masa* [59, 60].

**Table 7:** Effect of processing methods on colour attributes of buckwheat flour.

Samples	*L* ^∗^	*a* ^∗^	*b* ^∗^	Δ*E*
WBW	62.44 ± 0.01^c^	1.88 ± 0.01^c^	14.53 ± 0.01^b^	43.41 ± 0.01^c^
GBW	65.16 ± 0.59^b^	3.40 ± 0.05^a^	10.97 ± 0.14^d^	46.13 ± 0.59^b^
NNBW	68.51 ± 0.03^a^	3.39 ± 0.01^b^	12.42 ± 0.01^c^	49.48 ± 0.03^a^
SNBW	60.70 ± 0.01^d^	1.63 ± 0.01^d^	15.77 ± 0.01^a^	41.67 ± 0.01^d^

*Note:* Sample codes: WBW: Whole buckwheat flour, GBW: germinated buckwheat flour, NNBW: organically nixtamalized buckwheat flour; SNBW: synthetically nixtamalized buckwheat flour. *n* = 3. Means in the same column with the same superscripts are not significantly different (*p* > 0.05). Colour parameters: *L* ^∗^ represents lightness; *a* ^∗^ represents red‐green; *b* ^∗^ represents yellow‐blue; Δ*L* represents total colour difference (TCD).

### 3.8. Effect of Processing Methods on the Functional Properties of Buckwheat Flour

Table 8 summarises the results on the functional characteristics of the WBW, SNBW, GBW and NNBW. The WAC of NNBW (190.33 mL/g) was higher than that of the control (WBW) (74.67 mL/g); this could be attributed to the presence of a lower amount of hydrophilic constituents [61]. The high–water absorption capacity makes it desirable for use in cookies, sausage, bread and cakes. A similar trend was observed for OAC which ranged between 72.00 and 84.00 mL/g with NNBW having the highest (84.00 mL/g) and WBW the lowest (72.00 mL/g). Thus, natural nixtamalisation of buckwheat can improve both WAC and OAC, as revealed in this study.

**Table 8:** Functional properties of buckwheat flour as influenced by processing methods.

Samples	WAC (mL/g)	OAC (mL/g)	Swelling capacity (mL/g)	Solubility (mL/g)	Bulk density (g/mL)
WBW	74.67 ± 7.02^d^	72.00 ± 10.00^b^	9.59 ± 0.35^a^	42.00 ± 0.00^ab^	0.85 ± 0.04^a^
GBW	94.00 ± 3.61^c^	76.67 ± 4.04^ab^	9.87 ± 0.07^a^	52.67 ± 4.16^a^	0.80 ± 0.00^b^
NNBW	190.33 ± 1.16^a^	84.00 ± 2.65^a^	6.28 ± 0.58^c^	15.67 ± 3.21^c^	0.85 ± 0.02^a^
SNBW	149.00 ± 5.20^b^	82.67 ± 2.31^ab^	7.66 ± 0.09^b^	32.00 ± 12.00^b^	0.80 ± 0.00^b^

*Note:* Sample codes: NNBW: naturally nixtamalised buckwheat flour. *n* = 3. Means in the same column with the same superscripts are not significantly different (*p* > 0.05).

Abbreviations: GBW, germinated buckwheat flour; SNBW, synthetically nixtamalised buckwheat flour; WBW, whole buckwheat flour.

Usually, the high protein contents in flour and the nature of the proteins can contribute to the oil‐retaining properties of food materials [62]. It can therefore be stipulated that the higher OAC in NNBW suggested the presence of more polar amino acids than the control. Acid used in the process of nixtamalisation might have caused this. OAC is important in food product development, as it has influences on storage stability, particularly flavour binding and development of rancidity.

The swelling capacity of the flour samples ranges from 6.28 to 9.87 mL/g with GBW having the highest value (9.87 mL/g), while NNBW has the lowest value (6.28 mL/g). A similar trend was observed in the solubility of the flour samples ranging from 15.67 to 52.67 mL/g with GBW having the highest value (52.67 mL/g), while NNBW has the lowest value (15.67 mL/g). It can thereby be deduced that germination of buckwheat can enhance the swelling capacity and solubility of the resultant flour. A similar report was documented by Singh et al. [63]. However, a significant reduction in solubility and swelling power was observed in nixtamalised flour. The increased water solubility of flours was related to the release of water‐soluble compounds (i.e., the breakdown of macromolecules). Whereas, during germination, more hydrophilic moieties are exposed and bind to water owing to starch granule breakdown and changes in proteins. Mounika et al. [64] documented similar reports on the reduction of the swelling capacity of nixtamalised foxtail and maize flours. Food ingredients with good swelling capacity and solubility can be used in bakery products [61].

The BD of the samples ranged from 0.80 to 0.85 g/mL. NNBW had the highest value of BD, while GBW possessed the lowest. Both were higher than BD of the control sample, and it implied that both nixtamalisation and germination can improve the BD of buckwheat flour. The BD is a measure of the degree of coarseness of the sample. The observation in SNBW was similar to the report of Mounika et al. [64] during synthetic nixtamalisation of foxtail, maize grains and also in pearl millet flour [37]. However, Kumari et al. [65] reported that the application of the natural nixtamalisation processing method did not appear to have a significant impact on the BD of flour. The reduction in BD (both packed and loose) observed in GBW may be due to the breakdown of protein and starch which are complex compounds resulting in the modification that happened during germination [66].

## 4. Conclusion

The study revealed that germination and nixtamalisation processing methods improved the nutritional compositions (proximate composition [crude ash, fibre, protein and fat] and mineral contents [Mg, Na, K and Ca]), protein digestibility, antioxidant capacity (phenol, flavonoids, FRAP and total antioxidant), physicochemical (pH and TTA), colour attributes and functional properties (water and oil absorption capacities, swelling capacity, solubility and BD) of buckwheat flour. The processing methods also reduced the contents of antinutrients (phytate, tannin, saponin and oxalate) in the flour samples. Thus, nixtamalisation and germination can be used as veritable methods to enhance its value‐added end products in the marketplace and promote buckwheat flour utilisation.

## Conflicts of Interest

The authors declare no conflicts of interest.

## Funding

The authors did not receive any specific funding for this study.

## Data Availability

The data are included within the manuscript.
